# Frequency and types of clusters of major chronic diseases in 0.5 million adults in urban and rural China

**DOI:** 10.1177/26335565221098327

**Published:** 2022-05-20

**Authors:** Parisa Hariri, Robert Clarke, Fiona Bragg, Yiping Chen, Yu Guo, Ling Yang, Jun Lv, Canqing Yu, Liming Li, Zhengming Chen, Derrick A Bennett, on behalf of the China Kadoorie Biobank Collaborative Group

**Affiliations:** 1 Clinical Trial Service Unit and Epidemiological Studies Unit (CTSU), Nuffield Department of Population Health, 6396 University of Oxford, Oxford, UK; 2 Turku PET Centre, 8058 Turku University Hospital and University of Turku, Turku, Finland; 3 MRC Population Health Research Unit, Nuffield Department of Population Health, 6396 University of Oxford, Oxford, UK; 4 National Centre for Cardiovascular Diseases, Fuwai Hospital Chinese Academy of Medical Sciences, Beijing, China; 5 Department of Epidemiology and Biostatistics, School of Public Health, 12465 Peking University Health Science Center, Beijing, China

**Keywords:** Chronic disease, China, non-communicable disease, multimorbidity, frequency

## Abstract

**Background:**

Little is known about the frequency and types of disease clusters involving major chronic diseases that contribute to multimorbidity in China. We examined the frequency of disease clusters involving major chronic diseases and their relationship with age and socioeconomic status in 0.5 million Chinese adults.

**Methods:**

Multimorbidity was defined as the presence of at least two or more of five major chronic diseases: stroke, ischaemic heart disease (IHD), diabetes, chronic obstructive pulmonary disease (COPD) and cancer. Multimorbid disease clusters were estimated using both self-reported doctor-diagnosed diseases at enrolment and incident cases during 10-year follow-up. Frequency of multimorbidity was assessed overall and by age, sex, region, education and income. Association rule mining (ARM) and latent class analysis (LCA) were used to assess clusters of the five major diseases.

**Results:**

Overall, 11% of Chinese adults had two or more major chronic diseases, and the frequency increased with age (11%, 24% and 33% at age 50–59, 60–69 and 70–79 years, respectively). Multimorbidity was more common in men than women (12% vs 11%) and in those living in urban than in rural areas (12% vs 10%), and was inversely related to levels of education. Stroke and IHD were the most frequent combinations, followed by diabetes and stroke. The patterns of self-reported disease clusters at baseline were similar to those that were recorded during the first 10 years of follow-up.

**Conclusions:**

Cardiometabolic and cardiorespiratory diseases were most common disease clusters. Understanding the nature of such clusters could have implications for future prevention strategies.

## Introduction

Multimorbidity, involving the occurrence of two or more chronic diseases (where none is considered an index condition),^1^ is a growing public health problem worldwide. Improvements in access to health care and socioeconomic circumstances, in addition to epidemiological and demographic transitions, have resulted in significant improvements in life expectancy in low- and middle-income countries (LMICs), including China.^2^ However, these improvements in life expectancy have been accompanied by an increased prevalence of major chronic non-communicable diseases and multimorbidity.^3^

Multimorbidity is associated with a reduced quality of life,^4^ and results in substantial economic burden for healthcare systems.^5^ Studies in high-income countries (HICs) have reported that the both frequency of multimorbidity and the proportion of the population affected by multimorbidity have increased in recent years.^6^ However, the extent to which multimorbidity varies by age, sex and socioeconomic circumstances in LMICs, such as China, is uncertain.^7,8^

The leading causes of years of life lost (YLLs) in China in 2017 included stroke, ischaemic heart disease (IHD), lung cancer, chronic obstructive pulmonary disease (COPD) and liver cancer.^3^ The prevalence of cardiometabolic multimorbidity has doubled in recent years in China,^9^ possibly reflecting the high prevalence of established risk factors for cardiovascular disease (CVD) including hypertension, smoking in men and type 2 diabetes (T2D).^10^ Previous studies in Chinese adults have reported on the distribution of self-reported multimorbidity, but such studies have been constrained by reliance on self-reported diseases, small sample sizes^11^ or use of cross-sectional study designs.^4,10,12,13^

A systematic review of chronic disease multimorbidity in China reported that there was marked methodological heterogeneity among prevalence studies.^14^ A review of methodology found that clustering methods were the most widely used analytical approaches to identifying disease patterns in order to group together similar diseases that are found in the same individuals.^15^ For example, a recent paper used hierarchical agglomerative cluster analysis to assess multimorbidity patterns among a middle-aged population in Hong Kong.^16^ Association Rule Mining (ARM) is an alternative descriptive method that can reveal strong and frequent associations between diseases based on measures of ‘interestingness’ related to the effect sizes associated with particular patterns.^17^ As such, ARM can provide a different perspective to traditional clustering methods and has been utilized previously to assess multimorbidity in a Chinese population.^18^

We used data from the large nationwide China Kadoorie Biobank (CKB) prospective study to: (i) investigate the frequency of multimorbidity involving five major chronic diseases (stroke, IHD, COPD, T2D and cancer) overall and by age, sex, region and socioeconomic status; and (ii) assess how these five major diseases clustered in this population using a novel ‘association rules’ method in order to rank important disease associations which occur more often than would be expected by chance.

## Research design and methods

### Study population

Details of the CKB study design and methods of follow-up have been previously described.^19,20^ Briefly, the baseline survey was conducted between June 2004 and July 2008 in 10 diverse regions (five rural and five urban), chosen from China’s nationally representative Disease Surveillance Points (DSP) system. Regions were selected to maximize diversity in exposure and disease patterns, population stability and access to reliable evidence on death and disease incidence and local capacity. About 1.8 million people without major disability and aged 35–74 years were invited to participate in the study. Approximately 30% responded, and 512,726 individuals were enrolled, including 13,287 slightly outside the target age range of 30–79 years. Ethics approval was obtained from local, national and international ethics committees, and all participants provided written informed consent both for enrolment and for the follow-up study.

### Data collection

Trained local health workers collected information on sociodemographic factors, lifestyle (e.g. alcohol consumption, smoking, diet, physical activity) and prior medical history (including doctor-diagnosed IHD, stroke or transient ischaemic attack [TIA], cancer, COPD and diabetes) using interviewer-administered laptop-based questionnaires. Current use of medications for treatment of CVD was recorded for individuals who reported a prior history of IHD, stroke/TIA, hypertension or diabetes. Physical measurements, including blood pressure, lung function, height, weight and other anthropometric measures, were recorded using calibrated instruments and standard methods in all study regions. BMI was calculated as weight in kilograms divided by the square of the height in meters. A non-fasting venous blood sample was collected, for onsite random plasma glucose (RPG) testing using the Surestep plus meter (LifeScan, Milpitas, CA, USA), and remaining plasma was stored in liquid nitrogen. Data were collected on the time since the last meal. Individuals without self-reported diabetes and with an RPG level of 7.8–11.0 mmol/L were invited back the following day for a fasting plasma glucose (FPG) test. Prevalent diabetes was defined as a combination of self-reported and plasma glucose measurements above pre-specified cutoffs. Specifically, individuals with prevalent diabetes were defined as those with random plasma glucose (RPG) ≥7.0 mmol/L and time since last meal ≥ 8 h, or RPG ≥11.1 mmol/L and time since last meal <8 h or FPG ≥7.0 mmol/L on subsequent testing at baseline or self-reported diabetes at baseline

### Definition of multimorbidity

Major diseases for the present analyses were defined as the five major non-communicable diseases that accounted for most deaths in China, and included stroke, IHD, cancer, COPD and T2D.^21^ We included both self-reported diseases at enrolment (prevalent cases) and new-onset diseases during the follow-up period (incident cases), and the numbers for prevalent and incident diseases are shown in Webfigure 1. Multimorbidity was defined as presence of two or more of these five major diseases at baseline and during the first 10 years of follow-up. A recent systematic review of multimorbidity reported that these five diseases were among the top 20 causes of high disability adjusted life years or high years of life lost, of which four (cancer, coronary heart disease, stroke and diabetes) were among the top five causes.^22^

### Follow-up for morbidity and mortality

The status of study participants was monitored every 6 months by linkage to death registries, checked annually against local residential and health insurance records and through active confirmation by street committees and village administrators. The causes of death were coded by trained public health workers, blinded to baseline information, using the International Statistical Classification of Diseases and Related Health Problems, 10th revision (ICD-10). Data on hospital admissions were collected by electronic linkage, via each participant’s unique personal identification number, with established study-specific registries (for IHD, stroke, diabetes and cancer) and with the national health insurance reimbursement system for hospitalizations which provided almost universal coverage (∼97%) of study participants. Incident stroke, cancer, IHD, COPD and diabetes events were identified from the national health insurance system, disease registries and death certificates during follow-up until 31 December 2017. The ICD-10 codes are reported in Webtable 1. By 31 December 2017, 49,459 (9.6%) participants had died and 5302 (1.0%) were lost to follow-up.

### Statistical analysis

The mean values or proportions of baseline characteristics were estimated by occurrence of the five major diseases, standardized to the CKB population by 10-year age groups, sex and region, where appropriate. The frequencies of different combinations of these diseases were estimated for 10-year age groups, after adjustment for sex and region. Association Rule Mining (ARM), that uses a rule-based machine learning method, was used to examine the associations between individual diseases,^23^ and to identify combinations of the five major diseases.^24^ The derived association rules were visualized by graphical techniques using the R package arulesViz.^25^ The annotated values represent the diseases, and the arrows indicate the rules. The association rule yielded the ratios of observed/expected frequencies for each disease (the proportion of the dataset observed with a given comorbidity divided by the product of the prevalence of the individual diseases) to identify comorbidities that occur more often than would be expected by chance alone. This observed/expected ratio is referred to as the ***lift*** of an association rule and ***support*** is an indication of the frequency of each cluster of the five major chronic diseases. If the lift is > 1, this indicates that the two diseases were dependent on each other, and such rules were likely to be informative for predicting the risk of subsequent diseases. If the lift was < 1, this indicates that each of the two diseases had a negative effect on the presence of other disease and vice versa. The support and lift measures for each rule were displayed by the size and colour of the nodes. The support size was scaled for each plot individually (see eMethods for further details).

Sensitivity analyses were conducted after: a) excluding individuals with a prior history of stroke, IHD or cancer at baseline; and b) excluding participants with a prior history of any of the five major chronic diseases at baseline.

Additional sensitivity analyses utilized the more established Latent Class Analysis (LCA) in order to assess the concordance of the findings. For the CKB analyses of five major disease outcomes (stroke, cancer, IHD, COPD and diabetes), we conducted an LCA using the R package poLCA.^26^ LCA takes multiple diseases into account, and enables modelling of multiple comorbidities. Models were fitted without covariates with different numbers of pre-specified latent classes (between 2 and 5), with 5000 iterations to assess convergence of the model and the model was repeated 10 times with different starting values in order to assess the robustness of the estimation. The best fitting model was assessed via assessment of the Bayesian Information Criteria with the lowest value selected as this has been shown to be a more robust indicator of class enumeration.^27,28^ The likelihood ratio and Chi-square statistics were also utilized to assess model fit. Each latent class was labelled according to those chronic conditions whose prevalence exceeded the prevalence in the full cohort.^29^

After selection of an optimal model, each participant was assigned to the class for which they had the highest computed membership probability. An average posterior probability greater than 70% indicates an optimal fit.^30^ The characteristics of participants in different latent classes were compared using the chi-squared test for sex and region and ANOVA for age as a continuous variable. The statistical analyses were performed using SAS version 9.4 and R version 4.0.5.

## Results

### Population characteristics

Among the 512,726 participants, the overall mean (SD) age at baseline was 52 (10.7) years, 59% were women and 44% lived in urban areas. The mean (SD) levels of systolic blood pressure (SBP) and body mass index (BMI) were 131 (21) mmHg and 23.7 (3.4) kg/m^2^, respectively. At baseline, 74% of men and 3% of women reported smoking regularly, and 33% of men (*n* = 69 899) and 2% of women (*n* = 6245), respectively, reported drinking alcohol at least once each week (Webtable 2).

### Distribution of individual major chronic diseases

Stroke and IHD were the most frequent self-reported diseases at baseline and also accounted for the most frequent incident diseases occurring during follow-up. Overall, 13% (*n* = 65 025) of participants suffered a stroke, 12% (*n* = 63 164) had IHD, 10% (*n* = 50 570) had COPD, 10% (*n* = 49 194) had diabetes and 6% (*n* = 32 660) had cancer (Table 1). The frequency of all five major diseases increased linearly with increasing age, particularly for stroke, IHD and COPD. Stroke, cancer and COPD were more common among men than in women, but there were no sex-differences for IHD or diabetes.

**Table 1.** Frequency of major chronic diseases by baseline characteristics.

Characteristic	Cancer	Chronic obstructive pulmonary disease	Diabetes	Ischaemic heart disease	Stroke	Total population
Number of participants	32660 (6.4)	50570 (9.9)	49194 (9.6)	63164 (12.3)	65025 (12.7)	512,726
Age(years)						
30–39	1621 (2.2)	2807 (3.4)	2243 (3.1)	1991 (2.6)	1718 (2.1)	77,623
40–49	5869 (3.8)	7253 (4.9)	9973 (6.5)	9527 (6.1)	9338 (6.1)	152,769
50–59	10694 (6.8)	15018 (9.4)	17797 (11.3)	19785 (13.0)	20776 (13.8)	157,616
60–69	10003 (10.8)	16805 (17.9)	14027 (15.3)	21647 (23.4)	22404 (24.0)	91,753
70–79	4473 (13.1)	8687 (27.4)	5154 (15.1)	10214 (30.0)	10789 (30.7)	32,965
Mean (SD.)	57.9 (10.2)	59.1 (10.5)	56.9 (9.8)	59.4 (9.8)	59.7 (9.6)	52.0 (10.7)
Sex						
Men	15838 (7.2)	25077 (11.2)	18973 (8.9)	25496 (11.6)	30060 (13.6)	210,204
Women	16822 (5.7)	25493 (8.7)	30221 (10.2)	37668 (12.9)	34965 (12.0)	302,522
Area						
Rural	16537 (5.9)	33447 (12.1)	22663 (8.1)	29621 (10.8)	32193 (11.6)	286,533
Urban	16123 (6.9)	17123 (7.3)	26531 (11.3)	33543 (14.1)	32832 (13.8)	226,193
Highest education						
<6 years	18994 (6.8)	35482 (10.7)	27674 (10.1)	33722 (12.1)	36492 (13.6)	252,360
6+ years	13666 (6.1)	15088 (8.4)	21520 (9.7)	29442 (12.3)	28533 (12.0)	260,366
Annual household income, Yuan						
<20 000	18995 (6.6)	33641 (10.6)	26149 (9.5)	37843 (12.1)	41655 (13.2)	219,033
20 000+	13665 (6.2)	16929 (8.8)	23045 (10.0)	25321 (13.1)	23370 (12.4)	293,693

Values are n (%) or mean (SD) standardized to the age, sex and study region structure of the population, as appropriate.

Participants are classified as having a disease if present at the baseline survey or recorded during follow-up.

All diseases were more common in urban areas with the exception of COPD, which was more common in rural than urban areas. Most of the five diseases were also more common among those who had completed only primary education (<6 years). Except for diabetes and IHD, which were more common among people with an annual income greater than 20, 000 Yuan, all diseases were more common among participants with lower annual household income (Table 1**).** When the 10 regions were investigated separately, IHD (27%) and stroke (27%), were most frequent in Harbin, while COPD (22%) was most frequently diagnosed in Sichuan and diabetes (13%) and cancer (9%) were most frequent in Qingdao (Webfigure 2).

### Distribution of clusters of major chronic diseases

Overall, 2% (12,385) of participants had three or more of the major chronic diseases studied, 9% (45,237) participants had two diseases, 26% (131,441) participants had one disease and 63% (323,663) participants had none of the five diseases (Table 2). At baseline, participants with three or more major chronic diseases were older than those with none (63.5 [8.1] vs 48.8 [9.6] years, *p* < .001, respectively). After standardization for sex and region, only a few participants aged <60 years had any of the five diseases. Among participants aged 60–69 years, 37% had one disease, 18% had two diseases and 6% had three or more diseases. The highest proportion of participants aged 70–79 years at baseline had one disease (41%), one in four (25%) had two diseases and approximately one in ten (9%) had three or more diseases (Table 2).

**Table 2.** Baseline characteristics of participants by number of major chronic diseases.

	Number of diseases				
Characteristic	0	1	2	3+	Total population
Number of participants	323663 (63.1)	131441 (25.6)	45237 (8.8)	12385 (2.4)	512,726
Age (years**)**					
30–39	68165 (87.8)	8606 (11.1)	791 (1.0)	61 (0.1)	77,623
40–49	117497 (77.0)	29387 (19.2)	5121 (3.3)	764 (0.5)	152,769
50–59	93980 (59.1)	46516 (29.7)	14099 (9.2)	3021 (2.0)	157,616
60–69	35494 (39.1)	33860 (37.0)	16926 (18.2)	5473 (5.8)	91,753
70–79	8527 (26.5)	13072 (40.5)	8300 (24.5)	3066 (8.5)	32,965
Mean (SD.)	48.8 (9.6)	55.9 (10.2)	60.8 (9.2)	63.5 (8.1)	52.0 (10.7)
Sex					
Men	127144 (62.0)	56957 (26.4)	20557 (9.2)	5546 (2.5)	210,204
Women	196519 (64.1)	74484 (25.1)	24680 (8.5)	6839 (2.4)	302,522
Area					
Rural	185793 (63.9)	72830 (25.9)	22706 (8.3)	5204 (1.9)	286,533
Urban	137870 (62.2)	58611 (25.4)	22531 (9.4)	7181 (2.9)	226,193
Highest education					
<6 years	150632 (61.3)	75225 (26.9)	27267 (9.2)	7242 (2.5)	252,360
6+ years	173031 (64.8)	56216 (24.5)	17970 (8.3)	5143 (2.4)	260,366
Annual household income, Yu**an**					
<20 000	179295 (62.2)	78921 (26.3)	27958 (9.1)	7519 (2.4)	219,033
20 000+	144368 (63.5)	52520 (25.3)	17279 (8.7)	4866 (2.5)	293,693

Values are n (%) or mean (SD) standardized to the age, sex and study region structure of the population, as appropriate.

Participants are classified as having a disease if present at the baseline survey or recorded during follow-up.

**Figure 1.**
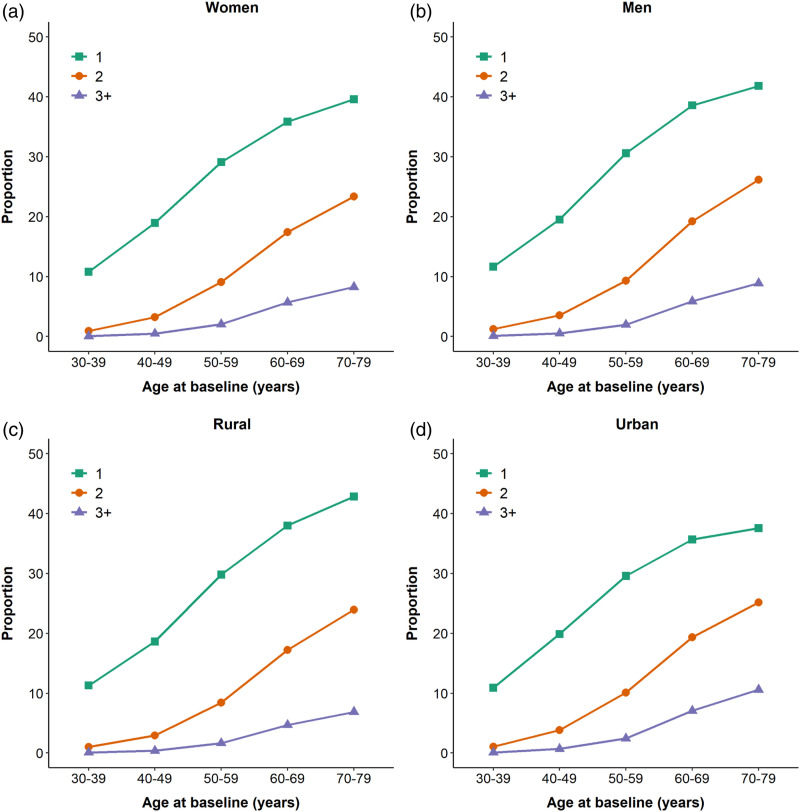
Proportion of major chronic diseases by age group, for each sex and area. The analyses were adjusted for region and sex where appropriate, including individuals with both prevalent and incident diseases.

**Figure 2.**
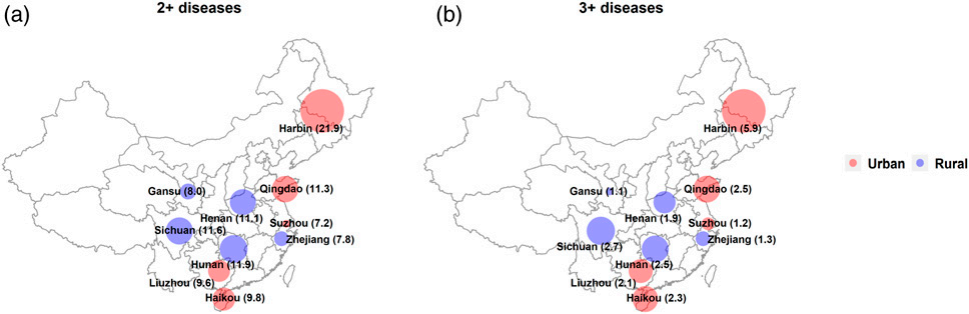
Proportion of participants with multimorbidity by study region. Numbers in parentheses represent percentage of participants with the disease combination. Numbers include individuals with both prevalent and incident diseases.

There were no differences between men and women in the frequency of major chronic diseases by age (Figure 1). The frequency of participants two or more chronic diseases was higher in urban than rural areas (9% vs 3%) as was the frequency of three or more conditions (8% vs 2%) (Table 2, Figure 1). Individuals who had completed only primary education had a higher frequency of two diseases, compared to those educated to middle-school level or higher (9% vs 8%). Participants whose annual income was less than 20 000 Yuan tended to have a similar frequency of two diseases as those with higher annual income (9% vs 9%). The proportions with two or more major diseases varied across the 10 study regions, ranging from 7% in Suzhou to 22% in Harbin, as did the proportion with three or more diseases (1% in Gansu to 6% in Harbin) (Figure 2).

When prevalent and incident diseases were investigated separately, the proportions of participants with one, two and three or more diseases also increased with increasing age. For incident diseases, the overall proportions of participants with at least one disease were higher than for the prevalent diseases (Webfigure 3). Among participants aged 70–79 years, the proportions for new onset versus existing diseases were: 5% vs <1% for three or more diseases, 17% vs 5% for two diseases and for one disease 43% vs 31% (Webfigure 3).

### Patterns and clustering of major chronic diseases

**Figure 3.**
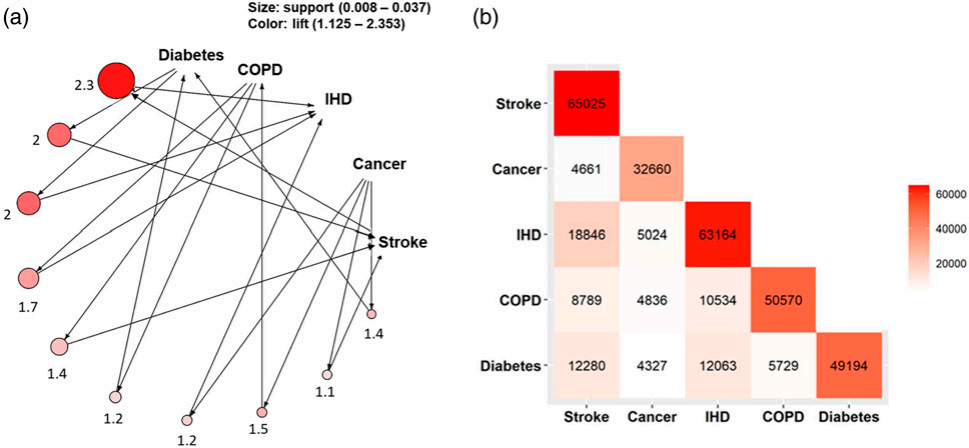
(a) Network and (b) heatmap of multimorbidity of two major chronic diseases. (a) The size of the circle represents the level of support associated with the rule and the colour shows the level of lift (the rule with the highest lift is coloured dark orange). The numbers represent the lift values for each pair of items. Support is a measure to identify the frequency of disease combinations in the dataset. Lift measures how many times more frequently two diseases occur together than expected if they were independent. (b) The numbers indicate the number of people having each pair of diseases. Numbers include individuals with both prevalent and incident diseases.

Figure 3(a) illustrates graphically the combinations of diseases derived using the ARM method and Webtable 3 provides the numerical details. The most frequent and strongest associations were found for IHD and stroke (support = 0.04, lift = 2.35, 95% CI [2.31–2.4]), followed by diabetes and IHD (support = 0.02, lift = 1.99, 95% CI [1.95–2.04]) and diabetes and stroke (support = 0.02, lift = 1.97, 95% CI [1.93–2.01]; Webtable 3). The support values suggest that overall, 4% of participants had multimorbidity related to stroke and IHD, 2% had multimorbidity based on diabetes and stroke, 2% had multimorbidity due to diabetes and IHD and 2% had multimorbidity due to COPD and IHD.

Among people from any age group, 2% had multimorbidity combinations of three diseases and <1% had multimorbidity combinations of four of the five major chronic diseases (Webtable 4). Patterns of disease clustering were similar among men and women (Webfigures 4 and 5). The frequency of multimorbidity was greater in older people. Among participants aged 60–69 years, the most frequent patterns of multimorbidity included the combinations of IHD and stroke (9%), diabetes and stroke (5%) and diabetes and IHD (5%), while among those aged 70–79 years they included IHD and stroke (12%) and COPD and IHD (9%) (Webtable 4).

### Sensitivity analyses

After excluding 25,514 individuals with self-reported diagnoses of cancer, IHD or stroke at baseline similar patterns were observed for frequency of disease clusters involving incident cases of major chronic diseases by age, sex and region (Webtable 5). Moreover, consistent with the overall population, stroke and IHD were the most common manifestations of multimorbidity (Webfigure 7). In further sensitivity analyses after excluding 84,289 individuals with any of the five major chronic diseases that were self-reported at baseline, the combinations of diseases and associations with age, sex and region were unaltered (Webtable 6, Webfigure 8).

The LCA model fits for all participants without exclusions for prior disease are summarized in Webtable 7. The model with 4 latent classes and the smallest BIC (1638937) was selected. These classes were labelled ‘mostly respiratory’, ‘mostly cardiometabolic’, ‘mostly diabetes’ and ‘relatively healthy’ based on the estimated probabilities of any particular chronic disease given membership of a latent class (Webtable 8).

The LCA model fits for all participants with exclusions for all prior diseases are summarized in Webtable 9. The model with 4 latent classes with the smallest BIC (1113601) was selected. These classes were labelled ‘mainly cancer’, ‘mostly cardiorespiratory’, ‘mostly cardiometabolic’ and ‘relatively healthy’ based on the estimated probabilities of any particular chronic disease given membership of a latent class (Webtable 10). For both analyses, the ‘relatively healthy’ group included those with a low prevalence of all of the five major chronic diseases; the ‘mostly cardiometabolic’ group was populated by those with stroke, IHD and diabetes; ‘mostly cardiorespiratory’ contained those participants with COPD, stroke and IHD.

The relationships with age, sex and region of study participants by latent class membership without and with exclusions are presented in Webtables 11 and 12, respectively. For all participants, the ‘relatively healthy group’ was youngest with an average age of 50.3 whilst the ‘mostly cardiometabolic group’ was oldest with an average age of 61.7 (Webtable 11). There were clear sex-differences with a higher prevalence of females for three of the four latent classes, which is consistent with the CKB population as a whole. The ‘mostly respiratory group included a higher proportion of in rural residents than urban (67.5% vs 32.5%), whereas the ‘mostly cardiometabolic’ group includes a higher proportion of urban residents (60.8% vs 39.2%, Webtable 11). There were also comparable differences by age, sex and region after excluding those with any of the prior diseases (Webtable 12). Overall, the LCA findings were broadly consistent with those of the main ARM analyses.

## Discussion

This large prospective study demonstrated that about 11% of Chinese adults aged 35–79 years had multimorbidity. The prevalence of multimorbidity varied from one quarter in individuals aged 60–69 years to one-third in participants aged 70–79 years. Multimorbidity was more common in those living in urban than in rural areas, and in individuals with lower levels of education. The highest frequency of multimorbidity was observed in residents of Harbin (an urban area in the north of China) and the lowest frequency was observed in Gansu (a rural area in the northwest of China). The simple descriptive analyses suggested that there were no sex-differences in the combinations of the five major chronic diseases considered in this report.

Comparisons with previous studies of the prevalence of multimorbidity have been complicated by differences in diagnostic criteria or definitions used to define multimorbidity (mainly reflecting differences in the number of diseases or the conditions investigated), the age-range of participants and populations studied.^31^ The present analyses used both prevalent and incident cases of five major chronic diseases, but the patterns were unaltered when the analyses were restricted to either prevalent or incident cases alone. The findings are consistent with those of a recent review of studies from LMIC and HICs that reported that multimorbidity was more common in individuals with lower levels of education and lower income.^32^ There were no differences between men and women in our simple descriptive analyses, but our ARM and LCA analyses found sex-differences similar to previous reports from China.^13^ However, this requires further investigation, including more diseases than those considered in the present study. A recent report from the China Health and Retirement Longitudinal Study, that used similar graphical methods to ours, but included more self-reported diseases at baseline, reported a higher prevalence of most major chronic diseases in women than in men, which they attribute to the longer life expectancy in women than in men.^33^

Previous population-based studies in China, investigating self-reported major chronic diseases, have reported a similar prevalence and pattern of multimorbidity with age and identified stroke and IHD as the most common disease combinations.^12^, ^6,13^ A previous CKB report that defined multimorbidity as the presence of two or more of 16 self-reported conditions at baseline found a higher prevalence among the elderly and those of lower socioeconomic status.^34^ Another CKB report investigated the impact of lifestyle factors (e.g. smoking, alcohol, diet, physical activity and body shape), on the development of cardiometabolic multimorbidity (CMM) and found that lifestyle played a role in transition from first cardiometabolic disease to CMM and to death.^35^

In the present report, the ARM analyses demonstrated that stroke and IHD were the most frequent combinations of multimorbidity. The analyses also identified diabetes and stroke, IHD and diabetes, COPD and IHD and COPD and stroke as the most frequent combinations in this population. These findings were supported by similar results from a latent class analysis. Other studies of multimorbidity (that included more diseases and used a different methodology) in HICs have also reported a high frequency of cardiorespiratory diseases,^36,37^ consistent with the findings of the present study.

The present study had several strengths, including large sample size, and a focus on well-characterized diseases involving a shared aetiology. The use of association rules enabled the evaluation of the strength of the relationships between different combinations of five major chronic diseases. The use of ARM facilitates assessment of the ranking of major chronic diseases which occurred more often than would be expected by chance alone. The ARM findings were also supported by LCA which also enabled comparisons of the estimated latent classes by age, sex and region. We also used a combination of incident and prevalent disease in our analyses in contrast to most previous studies which have restricted the definition of multimorbidity to prevalent diseases only.

However, this study had several limitations. Firstly, we only considered five major chronic diseases that have a high burden of disability adjusted life years and years of life lost in China. However, our analytical approach could easily be adapted to include a wider range of chronic diseases, and the present study serves as a useful exemplar of this analytical approach. Secondly, we did not consider risk factors for multimorbidity in this report. Given the definition of multimorbidity used, incorporating both self-reported disease at enrolment and incident disease during follow-up, investigation of the associations of individual risk factors (e.g. smoking, adiposity or blood pressure) with risk of multimorbidity would be constrained by the potential for reverse causality bias (whereby the onset of disease may be associated with changes in the levels of such risk factors). However, the inclusion of both prevalent and incident diseases ensured a comprehensive understanding of disease patterns and clustering. Moreover, the use of association rules to identify combinations of diseases is largely independent of reverse causality. Finally, the CKB is not representative of the Chinese population, although the associations of multimorbidity with age, income and education observed in the simple descriptive analyses used in present study are consistent with another nationally representative study from China that investigated multimorbidity using 14 diseases.^38^ Moreover, previous analyses have demonstrated the consistency between the results of the CKB and nationally representative surveys of the prevalence of certain chronic diseases.^39^

Overall, the higher frequency of multimorbidity based on five major chronic diseases in this Chinese population was positively correlated with age, differed by sex and region and was inversely related to duration of education. Cardiometabolic and cardiorespiratory diseases were identified as the most frequent disease clusters. The findings of the present study suggest that prevention strategies should target clusters of diseases, rather than individual diseases. Moreover, understanding disease clusters could inform appropriate therapeutic approaches and minimize risks of iatrogenic multimorbidity, for example, through use of medications that benefits some diseases but has an adverse effect on others.

## Supplemental Material

Supplemental Material - Frequency and types of clusters of major chronic diseases in 0.5 million adults in urban and rural China Click here for additional data file. Supplemental Material for Frequency and types of clusters of major chronic diseases in 0.5 million adults in urban and rural China by Parisa Hariri, Robert Clarke, Fiona Bragg, Yiping Chen, Yu Guo, Ling Yang, Jun Lv, Canqing Yu, Liming Li, Zhengming Chen, Derrick A Bennett and on behalf of the China Kadoorie Biobank Collaborative Group in Journal of Multimorbidity and Comorbidity.

## CKB Ethical approval

The China Kadoorie Biobank (CKB) complies with all the required ethical standards for medical research on human subjects. Ethical approvals were granted and have been maintained by the relevant institutional ethical research committees in the UK and China. Written informed consent was obtained from all participants included in the study.

## References for ethical approval

### UK

Kadoorie Study of Chronic disease in China (KSCDC) - Baseline, Oxford Tropical Research Ethics Committee (OxTREC) (2005) OxTREC Ref: 025-04

China Kadoorie Biobank (A prospective study of environmental and genetic causes of premature death in 500,000 Chinese adults) – Second resurvey (2013) OxTREC Reference: 1024–13

Objective measurement of physical activity in urban and rural Chinese: A feasibility study (2017) OxTREC Reference: 514–17

Assessing health risks associated with exposure to household and ambient air pollution in rural and urban China. (2017) OxTREC Reference: 5109–17

China Kadoorie Biobank – Third resurvey (2019) OxTREC Ref: 18–19

### China

Kadoorie Study of Chronic disease in China (KSCDC) – Baseline, Chinese Centre for Disease Control and Prevention. Ethical Review Committee (2004) Approval Notice 005/2004

Kadoorie Study of Chronic disease in China (KSCDC) – Second Phase, Chinese Academy of Medical sciences/Peking Union Medical College, Ethical Committee (2010) 24th December 2010

Kadoorie Study of Chronic disease in China (KSCDC) – Second Resurvey, Chinese Academy of Medical Sciences/Peking Union Medical College, Ethical Committee (2013) Ref: X1303262001
