# Supplementary material for: Frequency and types of clusters of major chronic diseases in 0.5 million adults in urban and rural China
Source: J Multimorb Comorb. 2022 May 20;12:26335565221098327. doi: 10.1177/26335565221098327 (PMC9125108; doi:10.1177/26335565221098327)
Supplement: Supplemental Material - Frequency and types of clusters of major chronic diseases in 0.5 million adults in urban and rural China [file sj-pdf-1-cob-10.1177_26335565221098327.pdf]

Supplementary material

**Frequency and types of cluster of five major chronic diseases among 0.5 million adults in urban and rural China**

Parisa Hariri^1,2^, Robert Clarke^1^, Fiona Bragg^1,3^, Yiping Chen^1,3^, Yu Guo^4^, Ling Yang^1^, Jun Lv^5^, Canqing Yu^5^, Liming Li^5^, Zhengming Chen^1,3^, Derrick A Bennett^1*^,
on behalf of the China Kadoorie Biobank Collaborative Group‡

1. Clinical Trial Service Unit & Epidemiological Studies Unit (CTSU), Nuffield Department of Population Health, University of Oxford, Oxford, UK
2. Turku PET Centre, Turku University Hospital and University of Turku, Turku, Finland
3. Medical Research Council Population Research Unit, Nuffield Department of Population Health, University of Oxford, Oxford, UK
4. Fuwai Hospital Chinese Academy of Medical Sciences, National Centre for Cardiovascular Diseases, Beijing, China
5. Department of Epidemiology and Biostatistics, School of Public Health, Peking University Health Science Center, Beijing, China.

*Corresponding author

‡Members of China Kadoorie Biobank collaborative Group are shown in the final page

**Contents list:**

[eMethods 4](#_Toc99432434)

[Webtable 1*.* ICD10 codes for the five major incident chronic diseases 6](#_Toc99432435)

[Webtable 2. Baseline characteristics of participants 7](#_Toc99432436)

[Webtable 3*.* Multimorbidity of pairs of major chronic diseases ordered by standardised lift values 8](#_Toc99432437)

[Webtable 4. Frequency of combinations of the five major chronic diseases by age at baseline 9](#_Toc99432438)

[Webtable 5. Frequency of the five major chronic diseases by baseline characteristics, excluding individuals with prevalent cancer, IHD or stroke 10](#_Toc99432439)

[Webtable 6. Frequency of the five major chronic diseases by baseline characteristics, excluding individuals with a prior of history of any of the five major chronic diseases at baseline 11](#_Toc99432440)

[Webtable 7. Estimation of the number of latent classes for the five major chronic diseases in all participants 12](#_Toc99432441)

[Webtable 8. Cluster/class response percentages within the five major diseases for all participants 13](#_Toc99432442)

[Webtable 9. Estimation of the number of latent classes for the five major chronic diseases with exclusions for prior disease 14](#_Toc99432443)

[Webtable 10. Cluster/class response percentages within the five major diseases with exclusions for prior disease 15](#_Toc99432444)

[Webtable 11. Assessment of the association of age, sex and region with latent class membership in all participants 16](#_Toc99432445)

[Webtable 12. Assessment of the association of age, sex and region with latent class membership in participants with exclusions for prior disease 17](#_Toc99432446)

[Webfigure 1. Flowchart of study population 18](#_Toc99432447)

[Webfigure 2. Proportion of participants with both prevalent and incident individual major chronic disease by study region 19](#_Toc99432448)

[Webfigure 3. Proportion of major chronic disease by (a) prevalent, (b) incident and (c) combined prevalent and incident cases by age group 20](#_Toc99432449)

[Webfigure 4 (a) Network and (b) heatmap of multimorbidity of pairs of major chronic diseases in men 21](#_Toc99432450)

[Webfigure 5 (a) Network and (b) heatmap of multimorbidity of pairs of major chronic diseases in women 22](#_Toc99432451)

[Webfigure 6. Flowchart of exclusions for the sensitivity analyses 23](#_Toc99432452)

[Webfigure 7. (a) Network and (b) heatmap of multimorbidity of pairs of major chronic diseases, excluding individuals with a prior history of cancer, IHD or stroke at baseline 24](#_Toc99432453)

[Webfigure 8. (a) Network and (b) heatmap of multimorbidity of pairs of major chronic diseases, excluding individuals with a prior history of any of the five major chronic diseases at baseline 25](#_Toc99432454)

[Members of the China Kadoorie Biobank collaborative group: 26](#_Toc99432455)

# **eMethods**

**Association rule mining**

The following metrics are used to identify the strength of the association rules:

- **Support:** The support value identifies the probability that two diseases A, B occur together in the dataset:

$$\mathrm{Support}\left( A,B \right)= P\left( A,B \right) .$$

- **Confidence**: The confidence shows how likely is that disease B occurs when disease A is already present:

$$\mathrm{Confidence}\left( A, B \right)=P\left( B | A \right)=\frac{P(A,B)}{P(A)} .$$

- **Lift:** The lift which also is called ratio of observed to expected, determines how many times more frequently two diseases A, B occur together than in isolation:

$$Lift(A,B)=\frac{P(A,B)}{P(A)P(B)} .$$

If lift is equal to 1, two diseases A, B are independent and have no association.

- **Standardized lift:** The range of possible values for the lift could differ from one rule to the other, therefore the interpretation of a lift value of say 2 which can be between (0,3) would be different from the lift value of 2 which is between (0,30). To overcome this problem we used standardized lift values introduced in, ^21^ using the following formula.

$$Std Lift \left( A, B \right)= \frac{Lift \left( A, B \right)- \frac{max\{P\left( A \right)+P\left( B \right)-1, 1/n\}}{(P\left( A \right)P\left( B \right))}}{\frac{1}{P\left( A \right)P\left( B \right)}- \frac{max\{P\left( A \right)+P\left( B \right)-1, 1/n\}}{(P\left( A \right)P\left( B \right))}}$$

Here n is the number of participants. Standardized lift takes a value between 0 and 1, and hence provides a fair comparison between the associations of diseases.

The confidence intervals for the lift values are calculated using so called ‘hyper-lift’ measure defined as

$hyper-{lift}_{\delta}(\left\{ A \right\}\to\{B\})=c_{AB}/Q_{\delta}(C_{AB})$.

Where $c_{AB}$ is the number of participants with two diseases$A, B$, and $Q_{\delta}(C_{AB})$ is the $\delta$ quantile of the hypergeometric distribution with parameters$c_{A},c_{B}$.

The $\left( 1-\alpha\right)=95\%$ confidence intervals for lift were calculated by

$[hyper-{lift}_{\frac{\alpha}{2}}, hyper-{lift}_{1-\frac{\alpha}{2}} ]$.

Mining association rules were performed using R package arules. Association rules were ranked by their standardized lift values. Because of the limited number of diseases we did not impose a lower threshold for the support and confidence, and listed all possible rules of length two.

**Latent Class Analysis**

Latent Class analysis (LCA) is used to identify patterns of responses to the indicator variables to create a set of mutually exclusive latent classes, that is, groups of individuals or other units of analysis. Individuals in the same latent class will have similar response patterns to the indicator variables whilst individuals across latent classes tend to have different response patterns to each other. In other words, LCA splits respondents into homogenous groups (latent classes). LCA was performed using the R package poLCA.

# **Webtable 1*.* ICD10 codes for the five major incident chronic diseases**

| **Disease** | **ICD 10 code** |
| --- | --- |
| Cancer | C00-C97 |
| Diabetes | E10-E14 |
| Ischaemic heart disease | I20-I25 |
| Stroke | I60-61 I63-64 |
| Chronic obstructive pulmonary disease | J41-J44 |

# **Webtable 2. Baseline characteristics of participants**

| **Characteristic** |  | **Men** | **Women** | **All** |
| --- | --- | --- | --- | --- |
| **Number of participants** | . | 210204 | 302522 | 512726 |
| **Area** | | | | |
| Rural | . | 118847 (56.6) | 167686 (55.2) | 286533 (55.9) |
| Urban | . | 91357 (43.4) | 134836 (44.8) | 226193 (44.1) |
| **Age , years** | | | | |
| 30-39 | . | 29566 (14.1) | 48057 (15.9) | 77623 (15.1) |
| 40-49 | . | 59218 (28.2) | 93551 (30.9) | 152769 (29.8) |
| 50-59 | . | 63751 (30.3) | 93865 (31.0) | 157616 (30.7) |
| 60-69 | . | 41340 (19.7) | 50413 (16.6) | 91753 (17.9) |
| 70-79 | . | 16329 (7.8) | 16636 (5.5) | 32965 (6.4) |
| Mean (SD) | . | 52.8 (10.9) | 51.4 (10.5) | 52.0 (10.7) |
| Education 6+ years | . | 121429 (59.3) | 130931 (42.2) | 252360 (49.2) |
| Annual household income 20 000+ yuan | . | 95937 (46.1) | 123096 (40.4) | 219033 (42.7) |
| Physical activity, MET-hours/day | . | 22.4 (15.3) | 20.2 (12.8) | 21.1 (13.9) |
| Ever regular smoking | . | 156287 (74.3) | 9797 (3.4) | 166084 (32.4) |
| Ever regular alcohol drinking | . | 69899 (33.6) | 6245 (2.1) | 76144 (14.9) |
| Systolic blood pressure | . | 132.4 (20.0) | 130.3 (22.0) | 131.1 (21.3) |
| Diastolic blood pressure | . | 79.2 (11.4) | 76.8 (10.9) | 77.8 (11.2) |
| Random plasma glucose | . | 59.5 (23.3) | 61.6 (23.4) | 60.7 (23.4) |
| BMI | . | 23.5 (3.2) | 23.8 (3.5) | 23.7 (3.4) |
| Waist to hip ratio | . | 0.9 (0.1) | 0.9 (0.1) | 0.9 (0.1) |
| Self-rated poor health | . | 18741 (8.8) | 34350 (11.5) | 53091 (10.4) |

Values are n (%) or mean (SD). MET h/day=metabolic equivalent of task hours per day. BMI=Body-mass index, kg/m²

# **Webtable 3*.* Multimorbidity of pairs of major chronic diseases ordered by standardised lift values**

| **Pairs of major chronic diseases** | **Count** |  | **Support** | **Confidence** | **Lift (95% CI)** | **Standardised Lift** |
| --- | --- | --- | --- | --- | --- | --- |
| IHD and stroke | 18846 |  | 0.0368 | 0.30 | 2.35 (2.31-2.4) | 0.29 |
| Diabetes and stroke | 12280 |  | 0.0240 | 0.25 | 1.97 (1.93-2.01) | 0.24 |
| IHD and diabetes | 12063 |  | 0.0235 | 0.19 | 1.99 (1.95-2.04) | 0.23 |
| COPD and IHD | 10534 |  | 0.0205 | 0.21 | 1.69 (1.65-1.73) | 0.20 |
| COPD and stroke | 8789 |  | 0.0171 | 0.17 | 1.37 (1.34-1.4) | 0.16 |
| Cancer and IHD | 5024 |  | 0.0098 | 0.15 | 1.25 (1.21-1.28) | 0.14 |
| Cancer and COPD | 4836 |  | 0.0094 | 0.15 | 1.5 (1.45-1.55) | 0.14 |
| Cancer and stroke | 4661 |  | 0.0091 | 0.14 | 1.13 (1.1-1.16) | 0.13 |
| Cancer and diabetes | 4327 |  | 0.0084 | 0.13 | 1.38 (1.34-1.43) | 0.12 |
| Diabetes and COPD | 5729 |  | 0.0112 | 0.12 | 1.18 (1.15-1.21) | 0.10 |

Support: a measure to identify the frequency of disease combinations in the dataset.

Confidence: how likely an individual is to have disease B when they already have disease A.

Lift: how many times more frequently diseases A and B occur together than expected if they were independent.

Standardised lift: ranks the association rules depending on the relative position of their lift to the maximum and minimum potential values of the lift.

Participants are classified as having a disease if present at the baseline survey or recorded during follow-up.

COPD= chronic obstructive pulmonary disease, IHD=ischaemic heart disease.

# **Webtable 4. Frequency of combinations of the five major chronic diseases by age at baseline**

|  | | **Age at baseline(years)** | | | | | |  |
| --- | --- | --- | --- | --- | --- | --- | --- | --- |
| **Disease combination** |  | | **30-39** | **40-49** | **50-59** | **60-69** | **70-79** | **All** |
| **A** | | | | | | | | |
| **Number of participants** | . | | 77623 | 152769 | 157616 | 91753 | 32965 | 512726 |
| **Combinations of two diseases** | | | | | | | | |
| Any two | . | | 791 (1.0) | 5121 (3.4) | 14099 (9.0) | 16926 (18.3) | 8300 (24.8) | 45237 (8.8) |
| IHD + stroke | . | | 188 (0.2) | 1682 (1.1) | 5152 (3.3) | 7754 (8.3) | 4070 (11.5) | 18846 (3.7) |
| Diabetes + stroke | . | | 146 (0.2) | 1307 (0.9) | 3934 (2.5) | 4801 (5.2) | 2092 (6.0) | 12280 (2.4) |
| Diabetes + IHD | . | | 170 (0.2) | 1231 (0.8) | 3726 (2.4) | 4791 (5.2) | 2145 (6.2) | 12063 (2.4) |
| COPD + IHD | . | | 114 (0.1) | 622 (0.4) | 2377 (1.5) | 4549 (5.0) | 2872 (9.0) | 10534 (2.1) |
| COPD +stroke | . | | 62 (0.1) | 487 (0.3) | 1948 (1.2) | 3799 (4.1) | 2493 (7.4) | 8789 (1.7) |
| COPD + diabetes | . | | 83 (0.1) | 506 (0.3) | 1706 (1.1) | 2326 (2.6) | 1108 (3.4) | 5729 (1.1) |
| Cancer + IHD | . | | 48 (0.1) | 426 (0.3) | 1311 (0.8) | 2105 (2.2) | 1134 (3.2) | 5024 (1.0) |
| Cancer + COPD | . | | 76 (0.1) | 341 (0.2) | 1191 (0.8) | 2018 (2.1) | 1210 (3.5) | 4836 (0.9) |
| Cancer + stroke | . | | 44 (0.1) | 386 (0.3) | 1285 (0.8) | 1903 (2.0) | 1043 (2.9) | 4661 (0.9) |
| Cancer + diabetes | . | | 70 (0.1) | 543 (0.4) | 1422 (0.9) | 1598 (1.7) | 694 (1.9) | 4327 (0.8) |
| **Combinations of three diseases** | | | | | | | | |
| Any three | . | | 52 (0.1) | 726 (0.5) | 2739 (1.7) | 4752 (5.1) | 2641 (7.6) | 10910 (2.1) |
| Diabetes + IHD + stroke | . | | 34 (0.0) | 350 (0.2) | 1267 (0.8) | 2051 (2.2) | 995 (2.8) | 4697 (0.9) |
| COPD + IHD + stroke | . | | 11 (0.0) | 101 (0.1) | 566 (0.4) | 1446 (1.6) | 985 (2.9) | 3109 (0.6) |
| COPD + diabetes + IHD | . | | 11 (0.0) | 79 (0.1) | 429 (0.3) | 851 (0.9) | 473 (1.4) | 1843 (0.4) |
| COPD + diabetes + stroke | . | | 6 (0.0) | 64 (0.0) | 337 (0.2) | 728 (0.8) | 408 (1.2) | 1543 (0.3) |
| Cancer + IHD + stroke | . | | 5 (0.0) | 74 (0.0) | 325 (0.2) | 694 (0.7) | 417 (1.1) | 1515 (0.3) |
| Cancer + diabetes + IHD | . | | 5 (0.0) | 64 (0.0) | 306 (0.2) | 485 (0.5) | 255 (0.7) | 1115 (0.2) |
| Cancer + diabetes + Stroke | . | | 5 (0.0) | 63 (0.0) | 253 (0.2) | 471 (0.5) | 212 (0.6) | 1004 (0.2) |
| Cancer + COPD+ IHD | . | | 4 (0.0) | 35 (0.0) | 159 (0.1) | 465 (0.5) | 303 (0.9) | 966 (0.2) |
| Cancer + COPD + stroke | . | | 3 (0.0) | 19 (0.0) | 144 (0.1) | 371 (0.4) | 261 (0.7) | 798 (0.2) |
| Cancer + COPD + diabetes | . | | 4 (0.0) | 35 (0.0) | 147 (0.1) | 278 (0.3) | 164 (0.5) | 628 (0.1) |
| **Combinations of four diseases** | | | | | | | | |
| Any four | . | | 9 (0.0) | 37 (0.0) | 271 (0.2) | 687 (0.7) | 403 (1.1) | 1407 (0.3) |
| COPD + diabetes + IHD + stroke | . | | 4 (0.0) | 17 (0.0) | 129 (0.1) | 338 (0.4) | 199 (0.6) | 687 (0.1) |
| Cancer + diabetes + IHD + stroke | . | | 2 (0.0) | 11 (0.0) | 98 (0.1) | 198 (0.2) | 104 (0.3) | 413 (0.1) |
| Cancer + COPD + IHD + stroke | . | | 2 (0.0) | 5 (0.0) | 35 (0.0) | 139 (0.1) | 105 (0.3) | 286 (0.1) |
| Cancer + COPD + diabetes + IHD | . | | 6 (0.0) | 40 (0.0) | 93 (0.0) | 58 (0.1) | . (0.2) | 197 (0.0) |
| **Combinations of five diseases** | | | | | | | | |
| All 5 diseases | . | | 1 (0.0) | 11 (0.0) | 34 (0.0) | 22 (0.0) | . (0.1) | 68 (0.0) |

Values are n (%) standardised to the sex and study region structure of the population.

Participants are classified as having a disease if present at the baseline survey or recorded during follow-up.

COPD= chronic obstructive pulmonary disease, IHD=ischaemic heart disease

# **Webtable 5. Frequency of the five major chronic diseases by baseline characteristics, excluding individuals with prevalent cancer, IHD or stroke**

| **Characteristic** |  | **Cancer** | **Chronic obstructive pulmonary disease** | **Diabetes** | **Ischaemic heart disease** | **Stroke** | **Total population** |
| --- | --- | --- | --- | --- | --- | --- | --- |
| **a** | | | | | | | |
| **Number of participants** | . | 28036 (5.8) | 20098 (4.1) | 31210 (6.4) | 45634 (9.4) | 51738 (10.6) | 487212 |
| **Age (years)** | | | | | | | |
| 30-39 | . | 1522 (2.0) | 483 (0.6) | 1681 (2.4) | 1800 (2.3) | 1629 (2.0) | 77277 |
| 40-49 | . | 5342 (3.5) | 1950 (1.4) | 7196 (4.8) | 8057 (5.2) | 8364 (5.5) | 150304 |
| 50-59 | . | 9266 (6.2) | 5833 (3.8) | 11804 (7.8) | 14755 (10.2) | 17011 (11.9) | 149767 |
| 60-69 | . | 8324 (10.1) | 7729 (9.0) | 8065 (9.9) | 14352 (17.9) | 16788 (20.5) | 81621 |
| 70-79 | . | 3582 (12.3) | 4103 (14.8) | 2464 (8.7) | 6670 (23.7) | 7946 (27.0) | 28243 |
| Mean (SD) | . | 57.4 (10.3) | 61.4 (9.3) | 55.7 (9.6) | 58.4 (10.1) | 59.0 (9.8) | 51.5 (10.5) |
| **Sex** | | | | | | | |
| Men | . | 13905 (6.7) | 10501 (4.8) | 11848 (5.9) | 18682 (8.9) | 23546 (11.2) | 199240 |
| Women | . | 14131 (5.0) | 9597 (3.5) | 19362 (6.9) | 26952 (9.7) | 28192 (10.1) | 287972 |
| **Area** | | | | | | | |
| Rural | . | 14778 (5.4) | 15788 (5.9) | 16664 (6.1) | 23901 (8.9) | 27326 (10.1) | 277064 |
| Urban | . | 13258 (6.2) | 4310 (2.0) | 14546 (6.8) | 21733 (10.0) | 24412 (11.2) | 210148 |
| **Highest education** | | | | | | | |
| <6 years | . | 16597 (6.2) | 15784 (4.6) | 18578 (6.5) | 25335 (9.3) | 29832 (11.3) | 239582 |
| 6+ years | . | 11439 (5.5) | 4314 (2.9) | 12632 (6.2) | 20299 (9.0) | 21906 (9.9) | 247630 |
| **Annual household income, yuan** | | | | | | | |
| <20 000 | . | 16292 (5.9) | 14049 (4.4) | 16002 (6.4) | 28008 (9.3) | 33575 (11.1) | 208478 |
| 20 000+ | . | 11744 (5.6) | 6049 (3.6) | 15208 (6.7) | 17626 (9.8) | 18163 (10.4) | 278734 |

Values are n (%) or mean (SD) standardised to the age, sex and study region structure of the population, as appropriate.

Participants are classified as having a disease if present at the baseline survey or recorded during follow-up, after excluding participants with cancer, IHD or stroke at baseline survey.

# **Webtable 6. Frequency of the five major chronic diseases by baseline characteristics, excluding individuals with a prior of history of any of the five major chronic diseases at baseline**

| **Characteristic** |  | **Cancer** | **Chronic obstructive pulmonary disease** | **Diabetes** | **Ischaemic heart disease** | **Stroke** | **Total population** |  |
| --- | --- | --- | --- | --- | --- | --- | --- | --- |
| **a** | | | | | | | | |
| **Number of Participants** | . | 23069 (5.4) | 11810 (2.8) | 16081 (3.8) | 36270 (8.5) | 41619 (9.7) | 428437 |  |
| **Age (years)** | | | | | | | | |
| 30-39 | . | 1448 (2.0) | 404 (0.5) | 1205 (1.8) | 1677 (2.2) | 1528 (2.0) | 73932 |  |
| 40-49 | . | 4874 (3.4) | 1448 (1.1) | 4429 (3.2) | 7228 (5.0) | 7413 (5.2) | 139837 |  |
| 50-59 | . | 7890 (6.1) | 3653 (2.8) | 6064 (4.5) | 12146 (9.7) | 14120 (11.4) | 130223 |  |
| 60-69 | . | 6358 (9.9) | 4225 (6.3) | 3435 (5.3) | 10589 (16.8) | 12757 (19.8) | 64035 |  |
| 70-79 | . | 2499 (12.0) | 2080 (10.8) | 948 (4.8) | 4630 (22.7) | 5801 (27.0) | 20410 |  |
| Mean (SD) | . | 56.4 (10.3) | 60.1 (9.8) | 54.0 (9.6) | 57.5 (10.1) | 58.2 (9.9) | 50.7 (10.3) |  |
| **Sex** | | | | | | | | |
| Men | . | 11092 (6.2) | 5850 (3.1) | 6119 (3.5) | 14608 (8.1) | 18682 (10.3) | 172823 |  |
| Women | . | 11977 (4.8) | 5960 (2.4) | 9962 (4.0) | 21662 (8.8) | 22937 (9.2) | 255614 |  |
| **Area** | | | | | | | | |
| Rural | . | 12260 (5.1) | 9305 (3.9) | 9333 (3.9) | 18991 (8.0) | 22296 (9.3) | 245113 |  |
| Urban | . | 10809 (5.8) | 2505 (1.3) | 6748 (3.6) | 17279 (9.1) | 19323 (10.2) | 183324 |  |
| **Highest education** | | | | | | | | |
| <6 years | . | 13244 (5.8) | 8996 (3.1) | 9642 (3.8) | 19392 (8.3) | 23491 (10.4) | 217472 |  |
| 6+ years | . | 9825 (5.1) | 2814 (2.1) | 6439 (3.5) | 16878 (8.2) | 18128 (9.0) | 210965 |  |
| **Annual household income, yuan** | | | | | | | | |
| <20 000 | . | 13192 (5.5) | 7953 (2.9) | 7921 (3.8) | 21945 (8.3) | 26937 (10.1) | 185768 |  |
| 20 000+ | . | 9877 (5.2) | 3857 (2.4) | 8160 (3.9) | 14325 (8.9) | 14682 (9.5) | 242669 |  |

Values are n (%) or mean (SD) standardised to the age, sex and study region structure of the population, as appropriate.

Participants are classified as having a disease if present during follow-up after excluding participants with any of the five diseases present at the baseline survey.

# **Webtable 7. Estimation of the number of latent classes for the five major chronic diseases in all participants**

| **Number of Latent Classes estimated** | **Residual degrees of freedom** | **Bayesian Information Criterion (BIC)** | **Likelihood ratio statistic** | **Chi-squared statistic** |
| --- | --- | --- | --- | --- |
| 2 | 20 | 1640195 | 1463.103 | 1558.357 |
| 3 | 14 | 1639184 | 372.944 | 390.97 |
| **4** | **8** | **1638937** | **47.528** | **47.614** |
| 5 | 2 | 1638972 | 2.851 | 2.848 |

Best fitting model with the lowest BIC is highlighted in bold font

# **Webtable 8. Cluster/class response percentages within the five major diseases for all participants**

| **Estimates class population shares** | **Class 1** | **Class 2** | **Class 3** | **Class 4** |
| --- | --- | --- | --- | --- |
|  | ***0.1746*** | ***0.1144*** | ***0.0514*** | ***0.6596*** |
| **Predicted class membership** | ***0.0886*** | ***0.0572*** | ***0.0648*** | ***0.7893*** |
| **Disease and item response probabilities** | | | | |
| Stroke | 0.11 | 0.49 | 0.15 | 0.07 |
| Cancer | 0.11 | 0.08 | 0.08 | 0.05 |
| Ischaemic heart disease | 0.15 | 0.56 | 0.08 | 0.04 |
| Chronic obstructive pulmonary disease | 0.62 | 0.16 | 0.00 | 0.02 |
| Diabetes | 0.10 | 0.26 | 0.94 | 0.00 |

Class 1: Mostly respiratory; Class 2: Mostly cardiometabolic; Class 3: Mostly diabetes; Class4: Relatively healthy

# **Webtable 9. Estimation of the number of latent classes for the five major chronic diseases with exclusions for prior disease**

| **Number of Latent Classes estimated** | **Residual degrees of freedom** | **Bayesian Information Criterion (BIC)** | **Likelihood ratio statistic** | **Chi-squared statistic** |
| --- | --- | --- | --- | --- |
| 2 | 11 | 1114191 | 769.9771 | 855.2064 |
| 3 | 14 | 1113619 | 119.0317 | 129.1991 |
| **4** | **8** | **1113601** | **23.16013** | **24.33908** |
| 5 | 2 | 1113659 | 3.324836 | 3.198483 |

Best fitting model with the lowest BIC is highlighted in bold font

# **Webtable 10. Cluster/class response percentages within the five major diseases with exclusions for prior disease**

| **Estimates class population shares** | **Class 1** | **Class 2** | **Class 3** | **Class 4** |
| --- | --- | --- | --- | --- |
|  | ***0.0264*** | ***0.1155*** | ***0.043*** | ***0.8150*** |
| **Predicted class membership** | ***0.0455*** | ***0.0322*** | ***0.0217*** | ***0.8996*** |
| **Disease and item response probabilities** | | | | |
| Stroke | 0.0479 | 0.2286 | 0.6516 | 0.0531 |
| Cancer | 0.9884 | 0.0907 | 0.0461 | 0.0207 |
| Ischaemic heart disease | 0.0148 | 0.3654 | 0.3723 | 0.0345 |
| Chronic obstructive pulmonary diseaseYes | 0.1220 | 0.2202 | 0.1004 | 0.0494 |
| Diabetes | 0.0457 | 0.0836 | 0.1411 | 0.0255 |

Class 1: Mainly Cancer; Class 2: Mostly cardiorespiratory; Class 3: Mostly cardiometabolic; Class 4: Relatively healthy

|  | Latent class group | | | |  |
| --- | --- | --- | --- | --- | --- |
| Characteristic | **Relatively Healthy** | **Mostly respiratory** | **Mostly cardiometabolic** | **Mostly diabetes** | **P-value for heterogeneity** |
| Age, mean (SE), years | 50.4 (0.016) | 58.5 (0.047) | 61.7 (0.059) | 55.0 (0.055) | <0.0001 |
| Sex, n (%) |  |  |  |  |  |
| Male | 162652 (40.2) | 22638 (49.8) | 12069 (41.2) | 12845 (38.6) | <0.0001 |
| Female | 242056 (59.8) | 22811 (50.2) | 17255 (58.8) | 20400 (61.4) |  |
| Region, n (%) |  |  |  |  |  |
| Urban | 176242 (43.6) | 14758 (32.5) | 17820 (60.8) | 17373 (52.3) | <0.0001 |
| Rural | 228466 (56.4) | 30691 (67.5) | 11504 (39.2) | 15872 (47.7) |  |
|  |  |  |  |  |  |

# **Webtable 11. Assessment of the association of age, sex and region with latent class membership in all participants**

# **Webtable 12. Assessment of the association of age, sex and region with latent class membership in participants with exclusions for prior disease**

|  | Latent class group | | | |  |
| --- | --- | --- | --- | --- | --- |
| Characteristic | **Relatively Healthy** | **Mainly cancer** | **Mostly cardiorespiratory** | **Mostly cardiometabolic** | **P-value for heterogeneity** |
| Age, mean (SD), years | 50.2 (0.016) | 56.1 (0.070) | 61.6 (0.082) | 59.6 (0.103) | <0.0001 |
| Sex, n (%) |  |  |  |  |  |
| Male | 162055 (40.1) | 10063 (49.2) | 7375 (49.0) | 3916 (40.6) | <0.0001 |
| Female | 242347 (59.9) | 10386 (50.8) | 7675 (51.0) | 5742 (59.4) |  |
| Region, n (%) |  |  |  |  |  |
| Urban | 170193 (42.1) | 9116 (44.6) | 5265 (35.0) | 5276 (54.6) | <0.0001 |
| Rural | 234209 (57.9) | 11333 (55.4) | 9785 (65.0) | 4382 (45.4) |  |
|  |  |  |  |  |  |

# **Webfigure 1. Flowchart of study population**

**
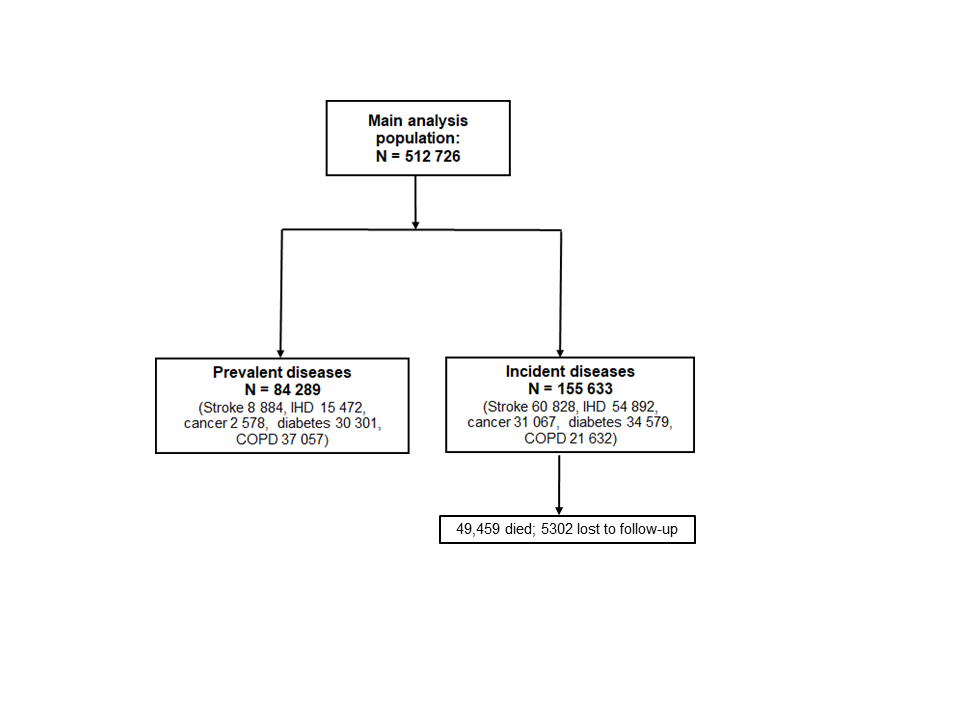
**

Prevalent disease refers to diseases present at baseline survey. Incident disease refers to diseases recorded during follow-up only.

# **Webfigure 2. Proportion of participants with both prevalent and incident individual major chronic disease by study region**


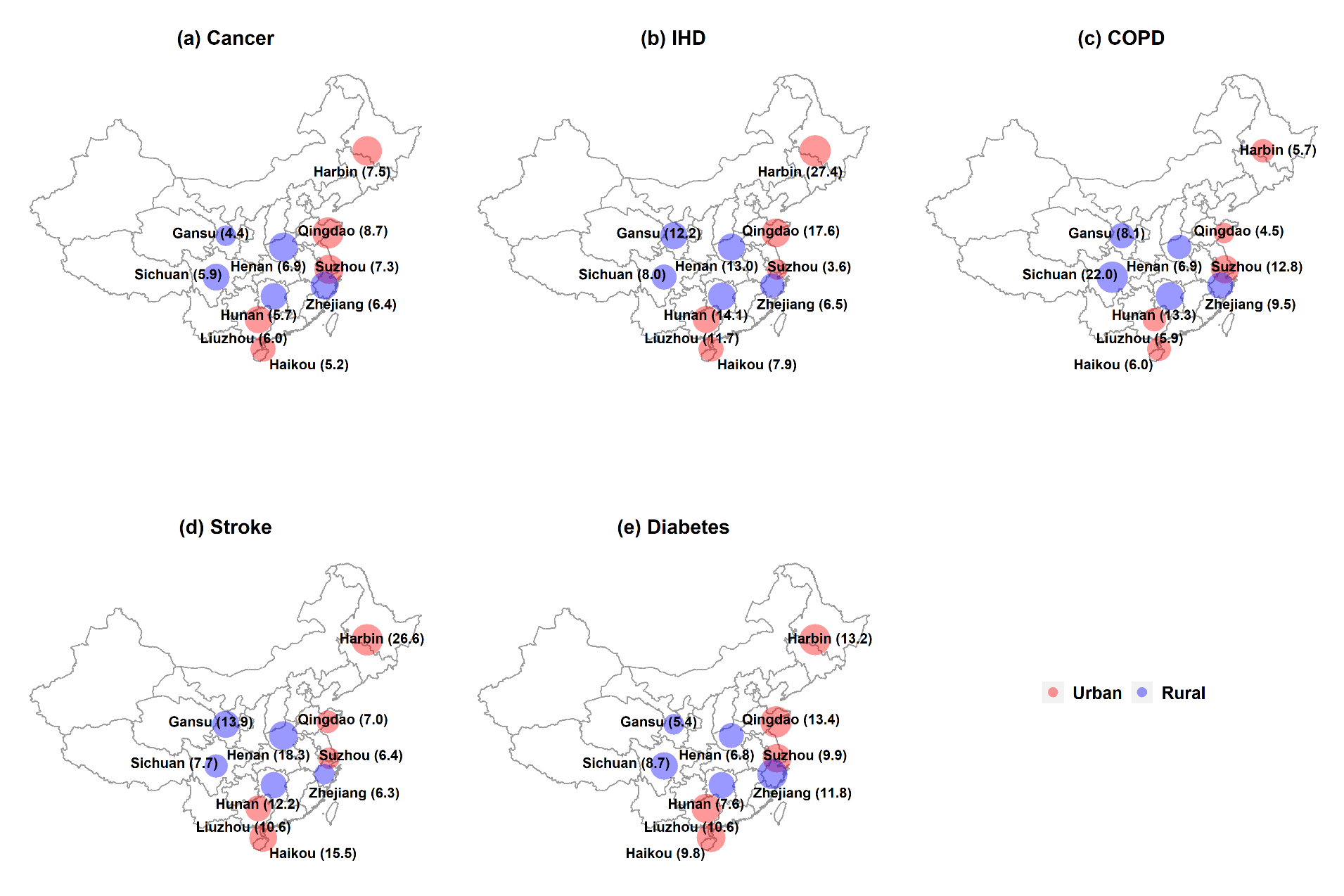


Numbers in parentheses represent of participants with diseases percentages. Participants are classified as having a disease if present at the baseline survey or recorded during follow-up. COPD= chronic obstructive pulmonary disease, IHD=ischaemic heart disease.

# **Webfigure 3. Proportion of major chronic disease by (a) prevalent, (b) incident and (c) combined prevalent and incident cases by age group**


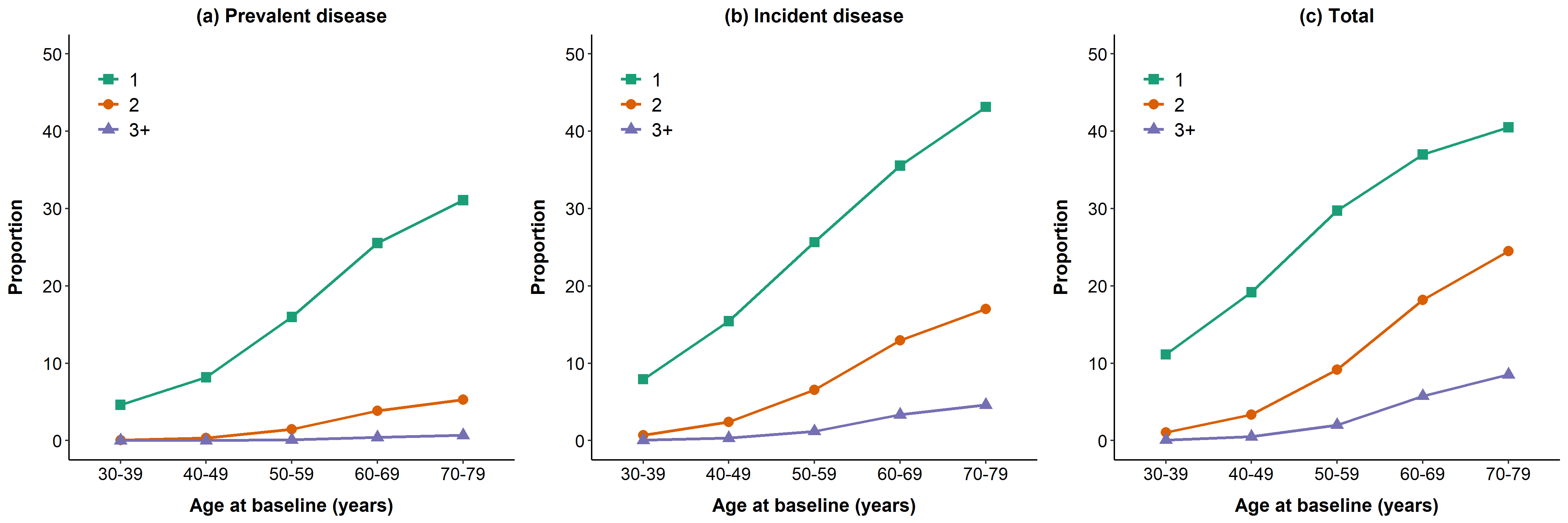


Prevalent disease refers to diseases present at baseline survey. Incident disease refers to diseases recorded during follow-up after excluding individuals with major chronic diseases at baseline survey.

# **
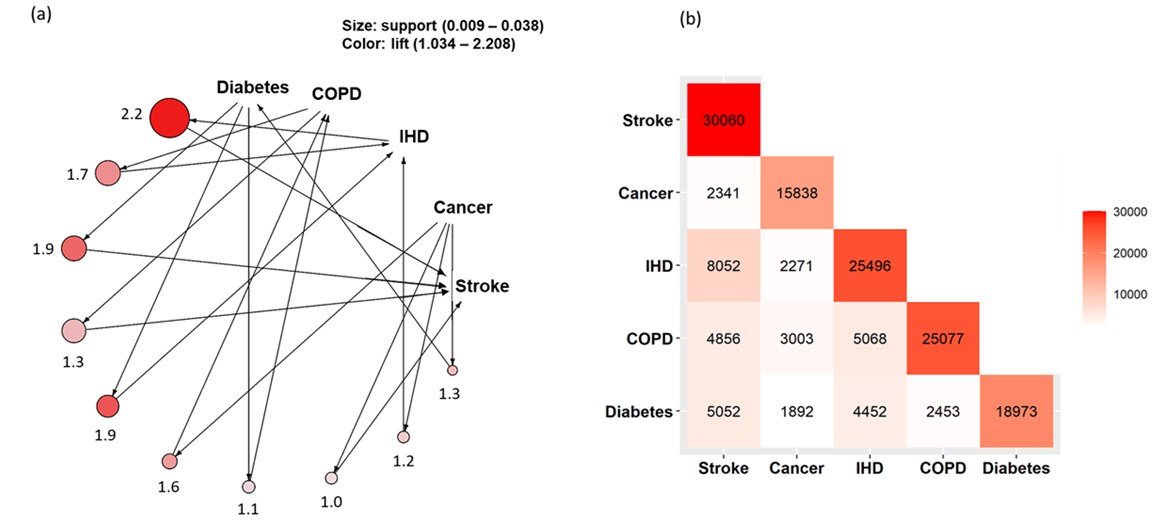
Webfigure 4 (a) Network and (b) heatmap of multimorbidity of pairs of major chronic diseases in men**

The size of the circle represents the level of support associated with the rule and the colour the level of lift (the rule with the highest lift is coloured dark orange). The numbers represent the lift values for each pair of items. Support is a measure to identify the frequency of disease combinations in the dataset. Lift measures how many times more frequently two diseases occur together than expected if they were independent.

(b) The numbers indicate the number of people having each pair of diseases.

Participants are classified as having a disease if present at the baseline survey or recorded during follow-up.

# **Webfigure 5 (a) Network and (b) heatmap of multimorbidity of pairs of major chronic diseases in women**


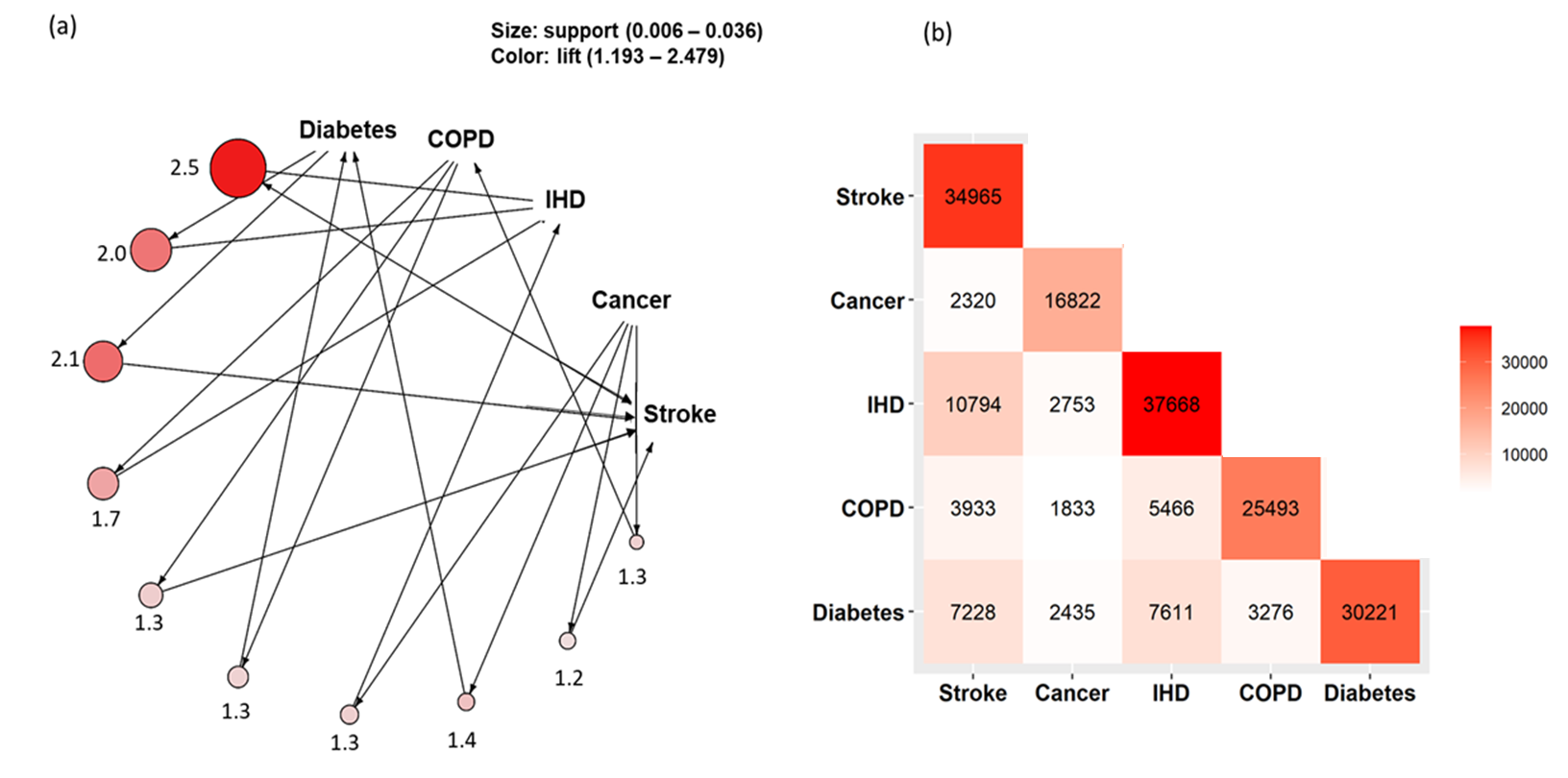


The size of the circle represents the level of support associated with the rule and the colour the level of lift (the rule with the highest lift is coloured dark orange). The numbers represent the lift values for each pair of items. Support is a measure to identify the frequency of disease combinations in the dataset. Lift measures how many times more frequently two diseases occur together than expected if they were independent.

(b) The numbers indicate the number of people having each pair of diseases.

Participants are classified as having a disease if present at the baseline survey or recorded during follow-up.

COPD= chronic obstructive pulmonary disease, IHD=ischaemic heart disease.

# **Webfigure 6. Flowchart of exclusions for the sensitivity analyses**


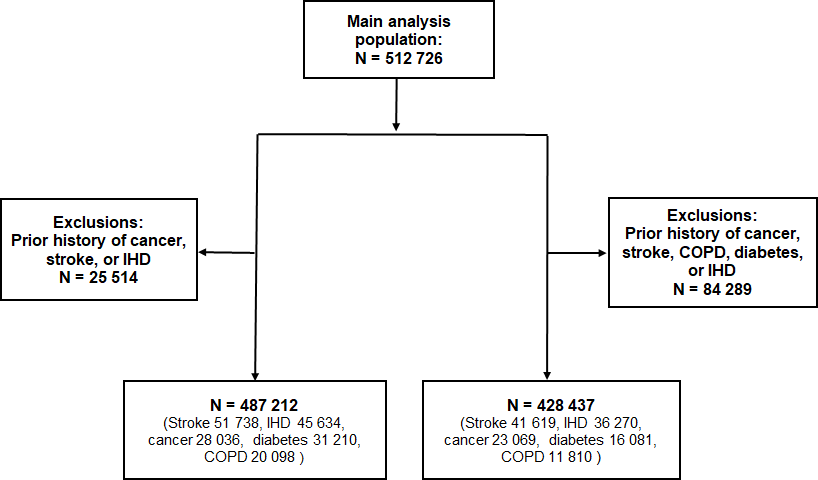


# **Webfigure 7. (a) Network and (b) heatmap of multimorbidity of pairs of major chronic diseases, excluding individuals with a prior history of cancer, IHD or stroke at baseline**


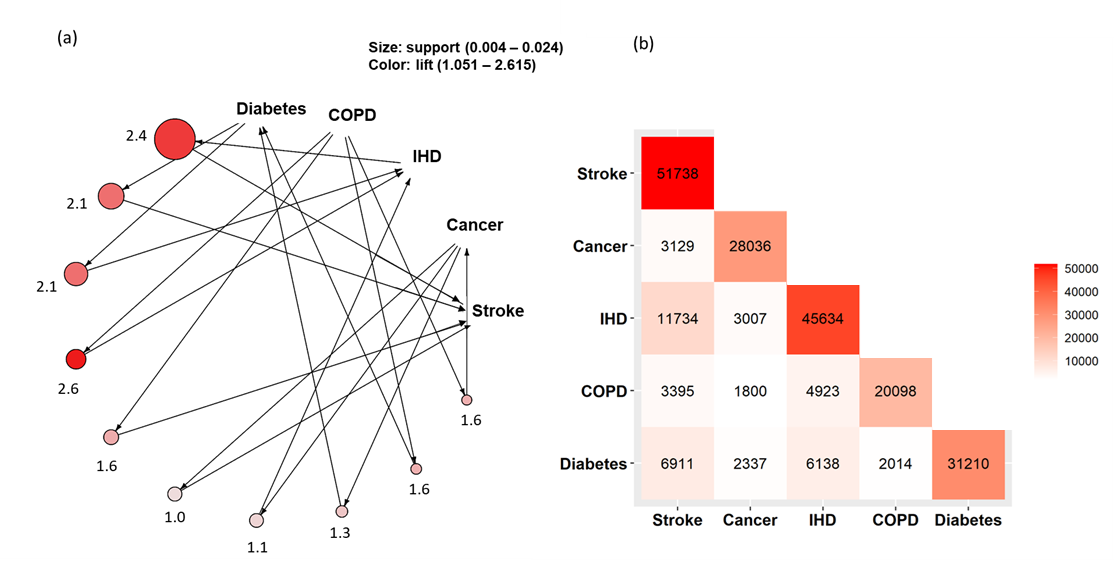


(a) The size of the circle represents the level of support associated with the rule and the colour shows the level of lift (the rule with the highest lift is coloured dark orange). The numbers represent the lift values for each pair of items. Support is a measure to identify the frequency of disease combinations in the dataset. Lift measures how many times more frequently two diseases occur together than expected if they were independent.

(b) The numbers indicate the number of people having each pair of diseases.

Participants with prior history of cancer, IHD or stroke were excluded.

COPD= chronic obstructive pulmonary disease, IHD=ischaemic heart disease.

# **Webfigure 8. (a) Network and (b) heatmap of multimorbidity of pairs of major chronic diseases, excluding individuals with a prior history of any of the five major chronic diseases at baseline**


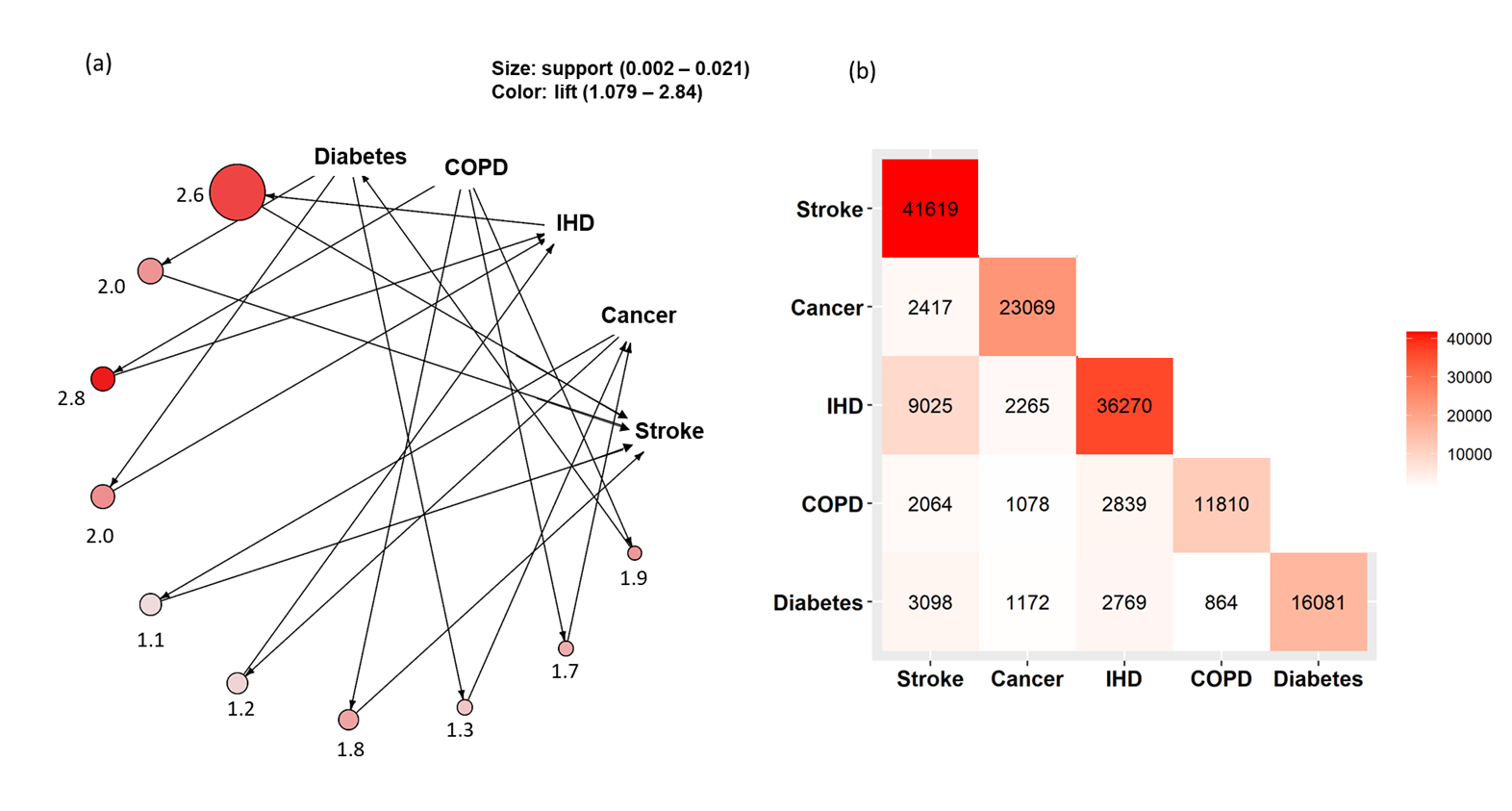


(a) The size of the circle represents the level of support associated with the rule and the colour shows the level of lift (the rule with the highest lift is coloured dark orange). The numbers represent the lift values for each pair of items. Support is a measure to identify the frequency of disease combinations in the dataset. Lift measures how many times more frequently two diseases occur together than expected if they were independent

(b) The numbers indicate the number of people having each pair of diseases

Participants with any of the five diseases present at the baseline survey were excluded

COPD= chronic obstructive pulmonary disease, IHD=ischaemic heart disease.

# **Members of the China Kadoorie Biobank collaborative group:**

**International Steering Committee:** Junshi Chen, Zhengming Chen (PI), Robert Clarke, Rory Collins, Yu Guo, Liming Li (PI), Chen Wang, Jun Lv, Richard Peto, Robin Walters.

**International Co-ordinating Centre, Oxford:** Daniel Avery, Fiona Bragg, Derrick Bennett, Ruth Boxall, Ka Hung Chan, Yumei Chang, Yiping Chen, Zhengming Chen, Johnathan Clarke; Robert Clarke, Huaidong Du, Zammy Fairhurst-Hunter, Hannah Fry, Simon Gilbert, Alex Hacker, Parisa Hariri, Mike Hill, Michael Holmes, Pek Kei Im, Andri Iona, Maria Kakkoura, Christiana Kartsonaki, Rene Kerosi, Kuang Lin, Mohsen Mazidi, Iona Millwood, Qunhua Nie, Alfred Pozarickij, Paul Ryder, Sam Sansome, Dan Schmidt, Paul Sherliker, Rajani Sohoni, Becky Stevens, Iain Turnbull, Robin Walters, Lin Wang, Neil Wright, Ling Yang, Xiaoming Yang, Pang Yao.

**National Co-ordinating Centre, Beijing:** Yu Guo, Xiao Han, Can Hou, Chun Li, Chao Liu, Jun Lv, Pei Pei, Canqing Yu.

**10 Regional Co-ordinating Centres:**

**Guangxi** Provincial CDC: Naying Chen, Duo Liu, Zhenzhu Tang. Liuzhou CDC: Ningyu Chen, Qilian Jiang, Jian Lan, Mingqiang Li, Yun Liu, Fanwen Meng, Jinhuai Meng, Rong Pan, Yulu Qin, Ping Wang, Sisi Wang, Liuping Wei, Liyuan Zhou. **Gansu** Provincial CDC: Caixia Dong, Pengfei Ge, Xiaolan Ren. Maiji CDC: Zhongxiao Li, Enke Mao, Tao Wang, Hui Zhang, Xi Zhang. **Hainan** Provincial CDC: Jinyan Chen, Ximin Hu, Xiaohuan Wang. Meilan CDC: Zhendong Guo, Huimei Li, Yilei Li, Min Weng, Shukuan Wu. **Heilongjiang** Provincial CDC: Shichun Yan, Mingyuan Zou, Xue Zhou. Nangang CDC: Ziyan Guo, Quan Kang, Yanjie Li, Bo Yu, Qinai Xu. **Henan** Provincial CDC: Liang Chang, Lei Fan, Shixian Feng, Ding Zhang, Gang Zhou. Huixian CDC: Yulian Gao, Tianyou He, Pan He, Chen Hu, Huarong Sun, Xukui Zhang. **Hunan** Provincial CDC: Biyun Chen, Zhongxi Fu, Yuelong Huang, Huilin Liu, Qiaohua Xu, Li Yin. Liuyang CDC: Huajun Long, Xin Xu, Hao Zhang, Libo Zhang. **Jiangsu** Provincial CDC: Jian Su, Ran Tao, Ming Wu, Jie Yang, Jinyi Zhou, Yonglin Zhou. Suzhou CDC: Yihe Hu, Yujie Hua, Jianrong Jin Fang Liu, Jingchao Liu, Yan Lu, Liangcai Ma, Aiyu Tang, Jun Zhang. **Qingdao** Qingdao CDC: Liang Cheng, Ranran Du, Ruqin Gao, Feifei Li, Shanpeng Li, Yongmei Liu, Feng Ning, Zengchang Pang, Xiaohui Sun, Xiaocao Tian, Shaojie Wang, Yaoming Zhai, Hua Zhang, Licang CDC: Wei Hou, Silu Lv, Junzheng Wang. **Sichuan** Provincial CDC: Xiaofang Chen, Xianping Wu, Ningmei Zhang, Weiwei Zhou. Pengzhou CDC: Xiaofang Chen, Jianguo Li, Jiaqiu Liu, Guojin Luo, Qiang Sun, Xunfu Zhong. **Zhejiang** Provincial CDC: Weiwei Gong, Ruying Hu, Hao Wang,Meng Wan, Min Yu. Tongxiang CDC: Lingli Chen, Qijun Gu, Dongxia Pan，Chunmei Wang, Kaixu Xie, Xiaoyi Zhang.

.
